# Depression and Risk of Delirium in Older Adults: A Retrospective Cohort Study Using German Primary Care Data

**DOI:** 10.3390/geriatrics11050127

**Published:** 2026-09-08

**Authors:** Christina Ryngler, Jens Bohlken, Nguyen Tien Huy, Karel Kostev

**Affiliations:** 1 Epidemiology, IQVIA, 06112 Halle, Germany; 2 Faculty of Medicine, Institute of Social Medicine, Occupational Health and Public Health, University of Leipzig, 04109 Leipzig, Germany; 3 Institute of Research and Development, Duy Tan University, Da Nang 50000, Vietnam; 4 School of Medicine and Pharmacy, Duy Tan University, Da Nang 50000, Vietnam; 5 School of Tropical Medicine and Global Health (TMGH), Nagasaki University, Nagasaki 852-8523, Japan; 6 Philipps-University, University Clinic, 35043 Marburg, Germany; 7 Epidemiology, IQVIA, 60597 Frankfurt, Germany

**Keywords:** depression, delirium, older adults, cohort study, primary care, dementia, propensity score matching

## Abstract

**Background:** Depression and delirium are common neuropsychiatric disorders in older adults. Although studies suggest an association between the two conditions, evidence regarding the long-term risk of delirium in older patients with depression in outpatient primary care remains limited. **Objective:** This study examined the relationship between depression and delirium in individuals aged ≥65 years. **Methods:** This retrospective cohort study used data from the German IQVIA Disease Analyzer database. It included individuals aged ≥65 years who received their first diagnosis of depression between 2005 and 2023. Using propensity score matching, patients with depression were matched 1:1 with individuals without depression. The primary outcome was incident delirium (ICD-10: F05). Time-dependent Cox regression models were applied for up to ten years of follow-up. **Results:** After matching, the study population consisted of 203,888 individuals (mean age: 75.8 years; 66% female). The incidence of delirium was higher in patients with depression than in controls (2.0% vs. 1.3%). Depression was associated with an elevated risk of delirium within the first two years after diagnosis (HR 1.42; 95% CI 1.14–1.75) and between six and ten years (HR 1.48; 95% CI 1.05–2.11). No statistically significant association was observed between years 3 and 5. Incident dementia was also associated with delirium (HR 1.52; 95% CI 1.14–2.02). **Conclusions:** Among older adults receiving outpatient care, depression was associated with a higher rate of subsequently documented delirium. These results complement existing evidence from inpatient settings. Due to the observational nature of the study, causal inferences cannot be made. In a sensitivity analysis excluding individuals with dementia at baseline, the association remained statistically significant across all intervals (HR 1.74, 1.53, and 1.82 for 0–2, 3–5, and 6–10 years, respectively).

## 1. Introduction

Delirium is an acute neuropsychiatric syndrome characterized by an acute onset and a fluctuating course of disturbances in attention, awareness and cognition [1]. It is one of the most common neuropsychiatric complications, particularly in older age, and is associated with functional impairments, cognitive decline, and increased morbidity and mortality [2,3]. A recent meta-analysis including 32 studies from Europe, North America, South America, Asia, and Oceania reported a pooled incidence of delirium of 28.8% among hospitalized older adults [3]. The development of delirium is understood as a multifactorial process in which predisposing and precipitating factors interact [2]. Factors consistently associated with delirium include older age, frailty, functional limitations, neurological diseases, sensory deficits, stroke, and substance use disorders, with cognitive impairment and dementia being the most consistently reported correlates [2,4,5].

Depressive disorders rank among the most disabling mental disorders worldwide [6]. Among older adults, the pooled global prevalence of major depressive disorder has been estimated at 13.3% [7]. In older age, depression frequently co-occurs with somatic illness and cognitive impairment, and newly diagnosed depression has been associated with a higher risk of subsequent dementia [8]. Given the central role of cognitive impairment in the development of delirium, an association between depression and delirium appears plausible.

For the relationship between depression and delirium, it should further be considered that depression in older age (“late-life depression”) may differ etiologically from depression with onset in younger or middle age. Late-life depression is characterized by a closer association with cerebral small-vessel disease, cognitive impairment, somatic comorbidity, and a poorer response to antidepressant treatment, whereas early-onset depression that persists into older age is more often associated with a positive family history of mood disorders and a lower vascular risk profile [9,10]. The very conditions associated with late-life depression—cerebrovascular pathology, cognitive decline, and dementia—are themselves among the established risk factors for delirium [2,5].

Several studies have reported an association between depression and delirium. Depression has been associated with a higher incidence of delirium in various clinical populations, including geriatric rehabilitation patients [11], very old, hospitalized patients [12], and patients undergoing cardiac surgery [13]. Associations between depressive symptoms and subsequent delirium have also been reported in prospective cohort studies and in a large community-based cohort [14,15]. The co-occurrence of both conditions has been linked to poorer outcomes, including functional decline, institutionalization, and higher mortality [16]. All these associations derive from observational data and therefore do not establish a causal relationship. Proposed shared pathways—including neuroinflammation, neurotransmitter imbalance, and dysregulated stress responses—are considered in detail in the Section 4.

Despite the growing body of evidence, most of the findings to date come from inpatient, surgical, or intensive care cohorts. Population-based studies on the long-term relationship between depression and delirium in older people in outpatient primary care are still rare. Large cohort studies that examine this relationship while taking into account relevant demographic and clinical influencing factors are lacking. Previous studies addressing depression and delirium in old age have largely taken the form of narrative reviews of the broader literature rather than primary analyses [17], and the largest available cohort analysis derives from the UK Biobank, a prospective community sample assembled through research assessment centers rather than routine outpatient records [15]. The present study differs from this prior work by using a retrospective, primary-care-based cohort drawn directly from German outpatient electronic medical records, with propensity score matching and up to ten years of time-dependent follow-up. The aim of the present study was therefore to investigate the association between depression and subsequently documented delirium in older adults in outpatient primary care.

## 2. Materials and Methods

### 2.1. Data Source

This retrospective cohort study is based on data from the Disease Analyzer database (IQVIA, Frankfurt am Main, Germany). The Disease Analyzer database has been used for epidemiological studies for many years and has already been used in numerous studies on depressive disorders [18,19,20]. It contains anonymized information on diagnoses, prescriptions and basic demographic characteristics of patients, which are collected from electronic medical records of outpatient medical practices [21].

The database includes approximately 3000 outpatient general and specialist practices in Germany, including general medical and specialist medical facilities. The selection of participating practices is based on statistics from the German Medical Association and takes into account factors such as specialty, federal state, municipality size and age of the treating physicians. This is intended to ensure the most representative picture possible of outpatient care in Germany [21].

Previous studies have shown that the Disease Analyzer database has a high degree of similarity with outpatient care in Germany with regard to documented patient and practice characteristics and is therefore suitable for population-based epidemiological analyses [21].

### 2.2. Study Population and Variables

**Figure 1:**
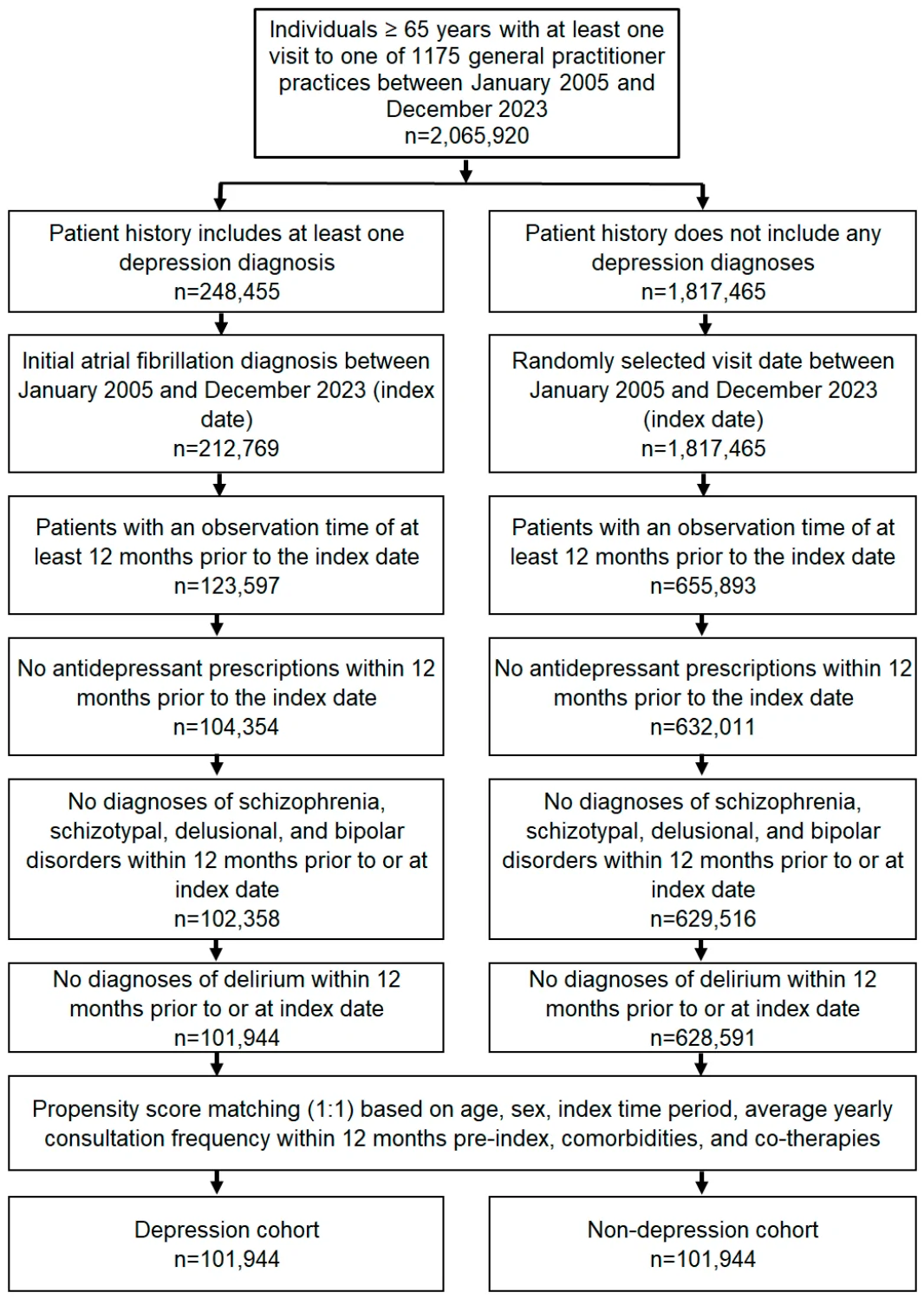
Selection of study patients.

This study included individuals aged 65 years or older with a depression diagnosis (ICD-10: F32, F33) in 1175 general practices in Germany between January 2005 and December 2023 (index date; Figure 1). A further inclusion criterion was an observation time of at least 12 months prior to the index date. Patients with schizophrenia, schizotypal, delusional (ICD-10: F20–F29), and manic or bipolar (ICD-10: F30, F31) or with delirium (ICD-10: F05) disorders documented within 12 months prior to or on index date were excluded. Patients with antidepressant prescriptions within 12 months prior to index date were also excluded. This criterion was based on prescription records within the database and cannot fully rule out prior antidepressant use documented elsewhere or discontinued earlier than 12 months before the index date; the depression cohort is therefore better described as free of recent antidepressant prescriptions rather than strictly treatment-naive. The index date was defined as the date of the first depression diagnosis (ICD-10: F32 or F33) documented in a patient’s entire available record; by this definition, no earlier depression diagnosis was present within the available observation period, and it marks the point of first clinical recognition of depression in outpatient care. Because documentation begins only when a patient enters a participating practice, a diagnosis established before the start of the available record cannot be ruled out (left truncation; see Section 4.4). Individuals in the comparison group had no documented depression diagnosis (F32/F33) at any point in their available record, before or after the index date. Prior delirium (F05), schizophrenia-spectrum (F20–F29), and manic/bipolar (F30, F31) disorders were ruled out over the entire pre-index record in the depression cohort and within the 12 months before the index date in the comparison cohort. Depression and delirium were ascertained exclusively from ICD-10 codes recorded during routine care rather than from standardized DSM-5 criteria, which may entail misclassification of late-life depression, hypoactive delirium, and coexisting cognitive impairment (see Section 4.4).

After applying similar inclusion criteria, individuals without depression were matched to depression patients using nearest neighbor propensity score matching (1:1). Covariates used for propensity score matching were selected a priori and included demographic characteristics, comorbidities, medication use, and healthcare utilization.

Demographic variables comprised age (continuous), sex, and index year (categorized into five periods: 2005–2008, 2009–2012, 2013–2016, 2017–2020, and 2021–2023).

Comorbidities were assessed based on documented diagnoses within five years prior to the index date (ICD-10 codes). These included hypertension (I10–I15), coronary heart disease (I20–I25), heart failure (I50), atrial fibrillation (I48), and cerebrovascular disease including stroke and transient ischemic attack (I63, I64, G45). Metabolic conditions comprised diabetes mellitus (E10–E14), obesity (E66), and lipid metabolism disorders (E78). Neurological disorders included dementia (F00–F03, G30), Parkinson’s disease (G20–G21), and epilepsy (G40–G41). Respiratory diseases included chronic obstructive pulmonary disease (J44) and asthma (J45). Renal and hepatic diseases were defined as chronic kidney disease (N18–N19) and chronic liver disease (K70–K77, B18), respectively. Malignant diseases were identified using all cancer diagnoses (C00–C97). Additional conditions included chronic pain disorders (M15–M19, M47–M48, M54), anxiety disorders (F40–F41), substance use disorders (F10–F19), sleep disorders (F51, G47), and personality disorders (F60–F69).

Medication use was assessed based on prescriptions within 12 months prior to the index date using Anatomical Therapeutic Chemical (ATC) classification codes. The following medication groups were considered: antipsychotics (N05A), antiepileptics (N03A), opioids (N02A), anticholinergic drugs (A03A, N04A, R06A), hypnotics and sedatives (N05C), corticosteroids (H02A, H02B), antihypertensive agents (C03A, C07A–C07B, C08A–C08B, C09A–C09D), antidiabetic drugs (A10), and anticoagulants (B01A, B01E, B01F).

Furthermore, the number of distinct active substances prescribed within 12 months prior to the index date was included as a continuous variable in the propensity score model. Healthcare utilization was measured as the number of outpatient visits within 12 months prior to the index date. The flow diagram of study participants is shown in Figure 1.

Standardized mean difference (SMD) was considered to examine the balance of covariate distribution between study cohorts after matching. In this study we allowed an SMD of less than 0.1 indicating that adequate covariate balance has been achieved [22,23]. Index year was included as one of several covariates in the propensity score model rather than as an exact-matching category, so the proportion of matched individuals within each five-year period was not required to be identical between cohorts as long as the overall SMD for this variable remained below 0.1, as observed in Table 1. Matching was performed as greedy nearest-neighbour 1:1 matching without replacement, using a caliper of 0.10 on the logit of the propensity score. All matching covariates were derived from structured diagnosis and prescription records and were complete—binary indicators were set to ‘absent’ when no corresponding code was documented—so that no imputation of missing covariate data was required. Index year was entered into the propensity score model as a single ordinal covariate; consequently, the overall standardized mean difference for index year was small even though the proportion of matched individuals differed across the individual five-year periods shown in Table 1.

### 2.3. Study Outcomes and Statistical Analyses

The outcome of the study was the first diagnosis of delirium (ICD-10: F05) during up to 10 years of follow-up after the index date. Descriptive statistics were used to summarize baseline characteristics of the study population. Continuous variables are presented as means and standard deviations or medians where appropriate, and categorical variables as proportions. Follow-up began at the index date and ended at the earliest of the first delirium diagnosis, the last documented practice contact (taken as the end of the observation period), or ten years after the index date. Because a reliable date of death was not available in the database, individuals who died were censored at their last recorded contact, and death could not be modelled as a competing event (see Section 4.4).

Time-to-event analyses were performed to investigate the association between depression and the incidence of delirium. Kaplan–Meier curves were used to estimate cumulative incidence.

Cox proportional hazards regression models were applied to estimate hazard ratios (HRs) with 95% confidence intervals (CIs). Initially, univariable Cox regression models were conducted to assess the crude association between depression and delirium.

The proportional hazards assumption was evaluated by including a time-dependent interaction term between exposure and log(time). This assumption was not met, indicating time-varying effects.

To account for the violation of the proportional hazards assumption, time-dependent Cox regression models were applied. Follow-up time was split into predefined intervals (0–2 years, 3–5 years, and 6–10 years), and separate exposure effects were estimated for each interval using time-varying covariates. These intervals were specified a priori to capture short-term (0–2 years), medium-term (3–5 years), and long-term (6–10 years) associations while retaining a sufficient number of delirium events in each interval for stable estimation.

In additional analyses, dementia was incorporated as a time-dependent covariate, defined as a diagnosis occurring during follow-up. This approach allowed differentiation between baseline risk and incident dementia during the observation period. In a further sensitivity analysis, the time-dependent Cox model was repeated after excluding all individuals with a documented dementia diagnosis at baseline, to examine whether the association also held in older adults free of pre-existing dementia.

Furthermore, exposure to antidepressant drug classes was included as time-dependent covariates. Antidepressants were categorized into tricyclic antidepressants (TCAs) (e.g., amitriptyline, clomipramine, imipramine, trimipramine, doxepin, opipramol), selective serotonin reuptake inhibitors (SSRIs), serotonin–norepinephrine reuptake inhibitors (SNRIs), and mirtazapine. For each class, treatment initiation during follow-up was modeled dynamically to account for changes in exposure over time.

Other antidepressant classes were not analyzed separately due to low prescription frequencies and the limited number of delirium events, which would have resulted in unstable estimates. Due to the relatively small number of delirium events, no age- or sex-stratified Cox regression analyses were conducted to avoid unstable estimates and limited statistical power.

All Cox regression models were stratified by matched pairs to account for the propensity score matching. Hazard ratios with 95% confidence intervals are reported. Statistical significance was defined as a two-sided *p*-value < 0.05.

Analyses were conducted using SAS version 9.4 (SAS Institute, Cary, NC, USA). This study is reported in accordance with the STROBE guideline and its RECORD extension for studies conducted using routinely collected health data.

## 3. Results

### 3.1. Characteristics of the Study Population

After propensity score matching, a total of 101,944 individuals with depression and 101,944 individuals without depression were included in the analysis. Baseline characteristics were well balanced between both groups, with all standardized mean differences below 0.1, indicating adequate matching (Table 1).

**Table 1:** Baseline characteristics of the study sample (after propensity score matching).

Variable	With Depression (%) N = 101,944	Without Depression (%) N = 101,944	SMD
Age (Mean, SD)	75.7 (6.9)	75.8 (7.0)	−0.005
Age ≤ 70	27,871 (27.3)	28,573 (28.0)	
Age 71–75	23,891 (23.4)	23,366 (22.9)	
Age 76–80	23,297 (22.9)	22,688 (22.3)	
Age > 80	26,885 (26.4)	27,317 (26.8)	
Women	67,046 (65.8)	67,612 (66.3)	0.012
Men	34,898 (34.2)	34,332 (33.7)	
2005–2008	7548 (7.4)	11,782 (11.6)	0.001
2009–2012	16,048 (15.7)	14,643 (14.4)	
2013–2016	26,437 (25.9)	21,796 (21.4)	
2017–2020	28,207 (27.7)	25,932 (25.4)	
2021–2023	23,704 (23.3)	27,791 (27.3)	
Number of physician visits within twelve months prior to index date (Median, IQR)	22 (39)	21 (39)	−0.001
Diseases documented within five years prior to index date			
Hypertension	57,029 (55.9)	57,872 (56.8)	−0.017
Ischemic heart diseases	21,552 (21.1)	21,554 (21.1)	0.000
Heart failure	12,378 (12.1)	12,521 (12.3)	−0.004
Atrial fibrillation and flutter	11,464 (11.3)	11,448 (11.2)	0.001
Cerebrovascular disease (ischemic stroke, unspecified stroke, and TIA; I63, I64, G45)	6948 (6.8)	7074 (6.9)	−0.005
Diabetes mellitus	25,597 (25.1)	25,885 (25.4)	−0.007
Obesity	8305 (8.2)	8371 (8.2)	−0.002
Dyslipidemia	33,092 (32.5)	33,423 (32.8)	−0.007
Dementia	6908 (6.8)	7212 (7.1)	−0.011
Parkinson’s disease (G20–G21)	1995 (2.0)	2056 (2.0)	−0.004
Epilepsy	1394 (1.4)	1402 (1.4)	−0.001
COPD	10,820 (10.6)	10,988 (10.8)	−0.005
Asthma	6640 (6.5)	6524 (6.3)	0.005
CKD	10,076 (9.9)	10,230 (10.0)	−0.005
Liver diseases	9353 (9.2)	9043 (8.9)	−0.011
Cancer	15,012 (14.7)	15,179 (14.9)	−0.005
Chronic pain/musculoskeletal disorders (osteoarthritis, spondylopathy, back pain; M15–M19, M47–M48, M54)	55,539 (54.5)	56,195 (55.1)	−0.012
Anxiety	6155 (6.0)	5425 (5.3)	0.030
Substance use disorders	1722 (1.7)	1671 (1.6)	0.004
Sleep disorders	15,244 (14.9)	14,727 (14.5)	0.014
Personality disorders	684 (0.7)	671 (0.7)	0.002
Drugs prescribed within twelve months prior to index date			
Antipsychotics	3904 (3.8)	3924 (3.9)	−0.001
Benzodiazepines	6573 (6.5)	6041 (5.9)	0.022
Antiseizure drugs	1288 (1.3)	1334 (1.3)	−0.004
Opioids	4379 (4.3)	4427 (4.3)	−0.002
Anticholinergics	5176 (5.1)	5180 (5.1)	0.000
Hypnotics	6573 (6.5)	6041 (5.9)	0.022
Corticosteroids	6305 (6.2)	6304 (6.2)	0.000
Antihypertensive drugs	60,079 (58.9)	60,684 (59.5)	−0.012
Antihyperglycemic drugs	14,402 (14.1)	14,531 (14.3)	−0.004
Anticoagulants	11,826 (11.6)	11,734 (11.5)	0.003
Number of different drugs (median, IQR)	5 (8)	4 (8)	−0.004

Proportions of patients in % given, unless otherwise indicated. SD: standard deviation; SMD: standardized mean difference; IQR: interquartile range; CKD: chronic kidney disease; COPD: chronic obstructive pulmonary disease.

The mean age was 75.7 years (SD 6.9) in patients with depression and 75.8 years (SD 7.0) in those without depression. Approximately two-thirds of the study population were women (65.8% vs. 66.3%). The distribution across age groups and index years was comparable between cohorts.

The median number of physician visits within 12 months prior to the index date was similar in both groups (22 vs. 21 visits). Likewise, the prevalence of comorbidities was highly comparable between groups, including hypertension (55.9% vs. 56.8%), ischemic heart disease (21.1% in both groups), diabetes mellitus (25.1% vs. 25.4%), and chronic back pain (54.5% vs. 55.1%).

Small differences were observed for some psychiatric conditions and medications, such as anxiety disorders (6.0% vs. 5.3%), benzodiazepine use (6.5% vs. 5.9%), and hypnotics (6.5% vs. 5.9%), but overall differences remained minimal. The median number of prescribed drugs was comparable between groups (5 vs. 4).

The median follow-up time was 4.3 years (IQR 1.9–8.1) in the depression cohort and 2.7 years (IQR 0.9–5.7) in the comparison cohort, corresponding to 545,814 and 394,421 person-years, respectively. A total of 1097 delirium diagnoses were documented in the depression cohort and 491 in the comparison cohort, corresponding to incidence rates of 2.0 and 1.2 per 1000 person-years. The longer observed follow-up in the depression cohort reflects more frequent and more continuous documented practice contacts, which extend the last recorded contact date; because all analyses are based on person-time (incidence rates per 1000 person-years and time-dependent hazard models), the comparison of delirium risk is not distorted by this difference in observation length.

### 3.2. Cumulative Incidence of Documented Delirium

**Figure 2:**
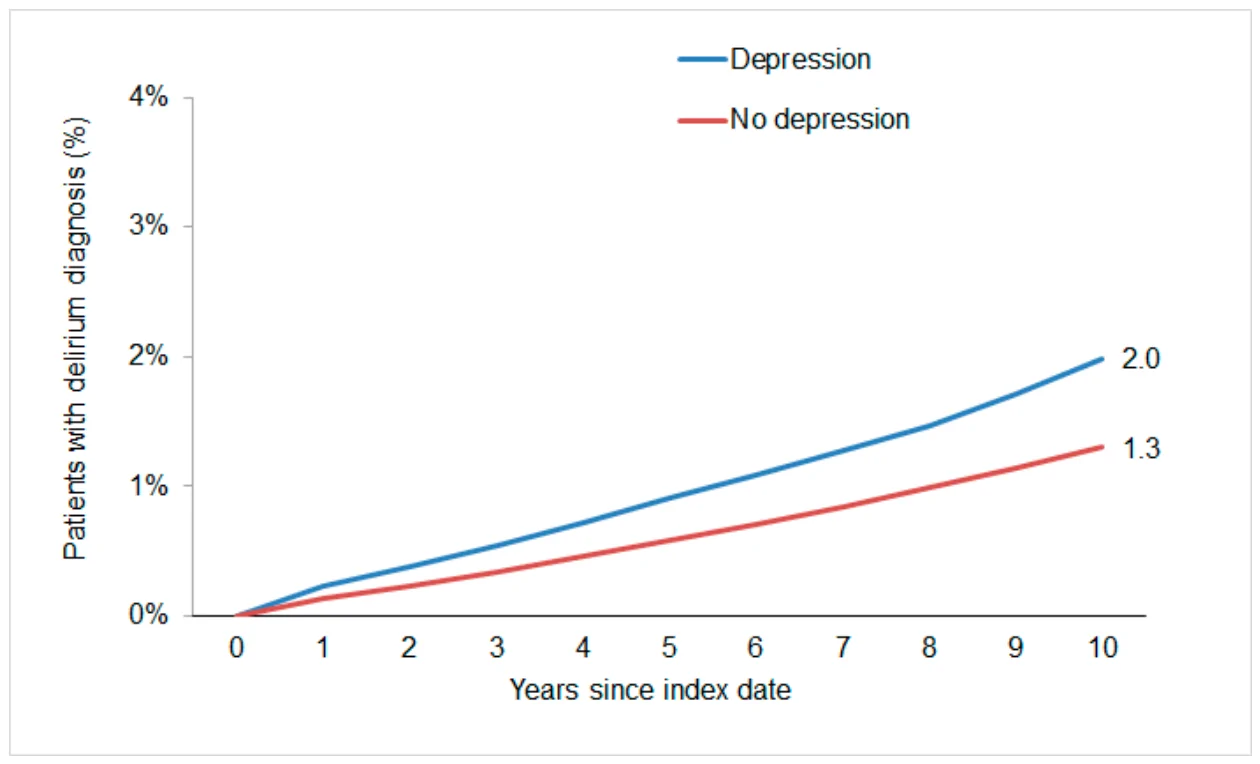
Cumulative incidence of delirium in individuals with and without depression.

During up to 10 years of follow-up, the cumulative incidence of delirium was higher among individuals with depression than among those without depression, reaching 2.0% and 1.3%, respectively (Figure 2).

### 3.3. Association of Depression with Subsequent Delirium Diagnosis

In univariable time-dependent Cox regression analyses, depression was associated with a higher incidence of delirium diagnosis across all time intervals. The corresponding hazard ratios were 1.62 (95% CI 1.33–1.98) within the first two years, 1.52 (95% CI 1.17–1.96) between three and five years, and 1.80 (95% CI 1.30–2.50) between six and ten years (all *p* ≤ 0.002) (Table 2).

In the multivariable time-dependent Cox regression model including dementia and antidepressant use as time-dependent covariates, the associations were attenuated but remained statistically significant in some intervals. Hazard ratios were 1.42 (95% CI 1.14–1.75; *p* = 0.002) within the first two years and 1.48 (95% CI 1.05–2.11; *p* = 0.027) between six and ten years, while the association between three and five years was not statistically significant (HR 1.21, 95% CI 0.91–1.61; *p* = 0.187) (Table 2).

Incident dementia during follow-up was associated with a higher likelihood of delirium (HR 1.52, 95% CI 1.14–2.02; *p* = 0.004). Regarding antidepressant classes, selective serotonin reuptake inhibitors (HR 1.69, 95% CI 1.19–2.40; *p* = 0.003) and mirtazapine (HR 1.52, 95% CI 1.05–2.18; *p* = 0.025) were associated with higher likelihoods of delirium, whereas tricyclic antidepressants (HR 0.82, 95% CI 0.58–1.16; *p* = 0.254) and serotonin–norepinephrine reuptake inhibitors (HR 1.20, 95% CI 0.62–2.32; *p* = 0.583) were not significantly associated.

The number of documented delirium events per interval was 336 (depression) versus 178 (comparison) within the first two years, 313 versus 153 between three and five years, and 448 versus 160 between six and ten years; the non-significant multivariable estimate for the 3–5-year interval therefore does not reflect a lack of events.

In the sensitivity analysis restricted to individuals without a documented dementia diagnosis at baseline, the association between depression and subsequent delirium remained statistically significant across all three intervals and was somewhat stronger than in the main analysis, with hazard ratios of 1.74 (95% CI 1.37–2.20) within the first two years, 1.53 (95% CI 1.15–2.04) between three and five years, and 1.82 (95% CI 1.30–2.57) between six and ten years (all *p* ≤ 0.004).

## 4. Discussion

### 4.1. Key Findings and Contextualization Within the Literature

In this population-based cohort study, older adults with a newly documented diagnosis of depression showed a higher incidence of subsequently documented delirium than matched individuals without depression. This association was also observed when incident dementia and antidepressant prescriptions during follow-up were taken into account. Given the observational design, these findings describe a statistical association and do not permit inferences about causality. Our findings are consistent with previous studies that have described the association between depression and delirium in various clinical contexts. A similar association has been observed in rehabilitation patients [11], very elderly hospitalized individuals [12], and cardiac surgery patients [13], as well as in population-based cohorts [14,15]. Notably, the association was statistically significant within the first two years and again between six and ten years, but not in the intervening period. This intermediate, non-significant estimate should be interpreted with caution. Because a substantial number of delirium events occurred within this interval (466 events; Section 3.3), it is unlikely to reflect limited statistical power alone; moreover, in the sensitivity analysis excluding individuals with baseline dementia, the association during the 3–5-year interval also reached statistical significance, suggesting that the intermediate attenuation in the main model may relate to the dynamics of coexisting dementia rather than a genuine absence of association. The present data do not allow a genuine attenuation of risk during this period to be distinguished from an estimate that lacked precision, and neither explanation can be confirmed with the available sample. More recent evidence is consistent with these observations: a longitudinal cohort study reported that the risk of subsequent dementia varies with the onset age of depression [24], and a recent systematic review confirmed that delirium is frequently underrecognized among older adults living in the community [25], underscoring the relevance of the present outpatient findings.

It is particularly noteworthy that the relationship between depression and delirium persisted in our study even after accounting for newly emerging dementia diagnoses. Since dementia is among the strongest known correlates of delirium [2,5], this suggests that the observed association is unlikely to be explained by neurodegenerative processes alone.

### 4.2. Possible Pathophysiological Mechanisms

The mechanisms underlying this relationship are not yet fully understood. Current models conceptualise delirium as the consequence of an increased vulnerability of the brain, determined by predisposing factors and acute precipitants [2,26]. Depression could increase this vulnerability and thereby lower the threshold at which an acute event culminates in delirium.

The inflammatory axis is particularly consistent with this assumption. Meta-analytic data show elevated C-reactive protein (CRP), interleukin-6, and tumour necrosis factor alpha (TNF-α) in patients with delirium [27], and the same markers are consistently elevated in depression [28]. Chronic low-grade inflammation in the context of depression may thus correspond to a state in which the ageing brain responds excessively to an acute somatic stimulus. A second pathway involves neurotransmission: a relative deficit of acetylcholine together with dopaminergic excess is among the most consistently described neurochemical models of delirium [26]. A third concerns the stress response. Chronic hyperactivity of the hypothalamic–pituitary–adrenal axis with sustained cortisol elevation is a well-characterised feature of depression, and the resulting glucocorticoid dysregulation interacts with inflammatory pathways to promote neuroinflammation [29]. This convergence of dysregulated stress signalling and inflammation offers one plausible link between the two conditions.

A neurodegenerative pathway must be distinguished from this: depression in old age is associated with a higher risk of subsequent dementia [8], and dementia is among the strongest correlates of delirium [2,5]. The fact that the association in our data persisted after incident dementia during follow-up was taken into account suggests that this pathway alone does not explain the observed pattern.

For the antidepressant findings, by contrast, no straightforward pharmacological mechanism can be inferred. The association of SSRI and mirtazapine use with more frequent documentation of delirium is most plausibly explained by confounding by indication, as treated patients are likely to have had more severe depressive symptoms. This is consistent with a recent review reporting a higher delirium risk after initiation of antidepressant therapy, whereas continuation of established SSRI therapy was associated with a lower risk [30], and with pharmacological data linking serotonergic antidepressant exposure to delirium duration rather than onset [31]. Owing to the observational design, causal statements about drug effects are not possible. These antidepressant-class analyses are therefore exploratory and hypothesis-generating and should not be interpreted as evidence of causal medication effects.

A notable finding is that tricyclic antidepressants, in contrast to SSRIs and mirtazapine, were not associated with an increased likelihood of delirium. At first glance, this result runs counter to pharmacological expectation, because tricyclic antidepressants carry the highest anticholinergic burden among the drug classes examined, and anticholinergic burden is one of the established pharmacological risk factors for delirium [26]. A plausible explanation lies in a channeling bias in prescribing: owing to their anticholinergic, cognitive, and cardiovascular risks, tricyclic antidepressants are listed as a drug class to be avoided in widely used criteria for potentially inappropriate medication in older adults and are therefore prescribed with restraint [32]. As a consequence, tricyclic antidepressants are preferentially prescribed to younger, less comorbid, and less vulnerable older patients, whereas precisely those patients who would be at higher baseline risk of delirium owing to frailty, multimorbidity, or incipient cognitive impairment less often receive tricyclic therapy. A comparable pattern has previously been described for other adverse effects of antidepressants in older age, with SSRIs and other newer antidepressants being associated with an increased risk of several adverse outcomes when directly compared with tricyclic antidepressants—a finding that the authors themselves attributed to channeling bias and residual confounding [33]. Our finding is therefore most plausibly interpreted as reflecting a guideline-concordant prescribing practice already based on risk stratification, rather than as evidence of a genuinely lower delirogenic potency of tricyclic antidepressants. This interpretation is further supported by the comparatively wide 95% confidence interval for tricyclic antidepressants, which points to a low prescribing frequency and thus limited statistical power in this subgroup.

### 4.3. Clinical Implications

These findings may be of clinical relevance, given that delirium is associated with increased morbidity, mortality, and use of health services [3,34].

Because the association was already apparent in outpatient primary care, general practitioners are well placed to recognize potentially vulnerable patients at an early stage. The findings suggest that depression may represent a potential marker of increased vulnerability to delirium in older adults. Whether established delirium prevention measures—such as regular medication reviews and prompt treatment of acute illness—are of particular benefit in this group cannot be answered by the present data and needs to be examined in dedicated studies [2].

### 4.4. Strengths and Limitations

Key strengths of this study include the large population-based sample, the use of a data source representative of outpatient care in Germany, the follow-up period of up to ten years, and the application of propensity score matching and time-dependent Cox regression models. A further strength is that individuals with any antidepressant prescription in the 12 months before the index date were excluded, which reduces the influence of prevalent antidepressant treatment on the observed associations. This exclusion criterion cannot, however, be equated with true treatment-naivety, because prescribing records in routine primary care may be incomplete and earlier antidepressant use outside the 12-month window cannot be excluded.

However, several limitations must be considered. Firstly, the identification of delirium was based on ICD-10 codes in routine data. Since delirium is often underreported in administrative data sets, delirium—particularly the hypoactive subtype—is frequently unrecognized in clinical practice and is known to be misclassified as depression, with low diagnostic concordance between non-psychiatric physicians and psychiatrists [35,36]. Since undetected delirium cannot be coded, an underestimation of the actual incidence is to be expected. This interpretation is consistent with the markedly lower cumulative incidence of delirium observed in this outpatient cohort (1.3% to 2.0% over up to ten years) compared with the pooled incidence of 28.8% reported for hospitalized older adults [3], although the two figures are not directly comparable given differences in setting, follow-up duration, and case ascertainment. Moreover, in patients already carrying a diagnosis of depression, hypoactive delirium may be attributed to the depressive disorder itself, which would lead to differential under-ascertainment in the exposed cohort and, if anything, bias our estimates towards the null. Secondly, no information could be considered regarding the severity or subtype of delirium or the severity of depression. Thirdly, potentially relevant influencing factors such as frailty, functional status, socioeconomic factors, or detailed measures of cognitive performance were not available. Therefore, residual confounding cannot be ruled out. Fourthly, approximately 7% of individuals in both cohorts had a documented dementia diagnosis prior to the index date (Table 1). Because pre-existing dementia is among the strongest known predisposing factors for delirium, its presence at baseline introduces a source of confounding that modeling incident dementia as a time-dependent covariate cannot fully address; to address this, a sensitivity analysis restricted to individuals free of baseline dementia was performed; the association between depression and delirium persisted and was, if anything, stronger in this subgroup (Section 3.3), indicating that it also holds in older adults without pre-existing dementia. Fifthly, because a reliable date of death was not recorded in the database, death could not be modelled as a competing event; given the advanced age of the cohort and the long follow-up, the Kaplan–Meier method may overestimate the cumulative incidence of delirium, whereas the incidence-rate and hazard-based estimates are less affected by this limitation. Sixthly, the analyses of individual antidepressant classes are exploratory and subject to confounding by indication and therefore do not permit conclusions about causal drug effects. Seventhly, prior delirium was ruled out over the entire pre-index record in the depression cohort but only within the 12 months before the index date in the comparison cohort; earlier delirium episodes among comparison individuals can therefore not be fully excluded, which may lead to some misclassification of incident delirium in this group.

Two further points regarding potential bias should be noted. The direction of ascertainment bias is uncertain: whereas undetected hypoactive delirium would bias the estimates towards the null, closer clinical and psychiatric attention to patients already diagnosed with depression could increase the likelihood that delirium is recognized and coded, biasing the estimates in the opposite direction; matching on the number of prior physician visits was intended to mitigate, but cannot fully exclude, such differential ascertainment. In addition, depression was identified from routine diagnostic codes without a required confirmatory second diagnosis, so that some degree of exposure misclassification cannot be ruled out. The longer average follow-up in the depression cohort is a further facet of this differential-ascertainment concern, since a longer observation period offers more opportunity for delirium to be detected and coded; the person-time–based analyses reduce but cannot entirely remove this effect.

A further aspect to consider is the etiological heterogeneity of the diagnosis of depression in older age. In addition to classic affective depression, late-life depression also encompasses forms that are predominantly co-determined by cerebral small-vessel disease, microvascular dysfunction, and metabolic risk factors, and—particularly with late onset—is more frequently accompanied by cognitive impairment and structural brain changes [10,37,38]. Because administrative diagnostic codes (ICD-10: F32, F33) do not distinguish between these etiologically distinct subtypes, it is conceivable that part of the observed association between depression and delirium is attributable to an underlying cerebrovascular or neurodegenerative vulnerability that is already captured by the diagnosis of depression itself—rather than only by separately recorded incident dementia [2,5]. This diagnostic imprecision represents an additional limitation that is independent of the misclassification of the delirium outcome. Because the index date reflects the first recorded diagnosis of depression rather than its first lifetime onset, the present design cannot distinguish late-onset depression from early-onset depression persisting into old age; the distinction between early- and late-life depression outlined in the Introduction therefore provides conceptual and pathophysiological background rather than a hypothesis directly tested in this study.

### 4.5. Future Research Directions

Future research should examine whether established delirium-prevention measures—such as regular medication review and prompt treatment of acute illness—are of particular benefit in older adults with depression, ideally in prospective studies that incorporate delirium subtype and severity, depression severity, frailty, and detailed cognitive measures, and that model death as a competing risk. Linkage of primary-care data with hospital and mortality records would further improve the ascertainment of delirium and of competing events.

## 5. Conclusions

Older adults with newly diagnosed depression had a higher rate of subsequently documented delirium in outpatient primary care. This association remained after consideration of incident dementia and antidepressant treatment during follow-up. While no causal conclusions can be drawn, these findings indicate that depression may identify a subgroup of older adults in whom delirium is documented more frequently and who may warrant increased clinical attention. Further studies are needed to clarify the mechanisms underlying this association.

## Figures and Tables

**Table 2:** Association between depression and subsequent delirium diagnosis in patients followed in general practices in Germany (time-dependent Cox regression models).

Time Period/Variable	Univariable Time-Dependent Cox Regression Model		Multivariable Time-Dependent Cox Regression Model	
	HR (95% CI)	*p* Value	HR (95% CI)	*p* Value
≤2 years	1.62 (1.33–1.98)	<0.001	1.42 (1.14–1.75)	0.002
3–5 years	1.52 (1.17–1.96)	0.002	1.21 (0.91–1.61)	0.187
6–10 years	1.80 (1.30–2.50)	<0.001	1.48 (1.05–2.11)	0.027
Dementia			1.52 (1.14–2.02)	0.004
TCA			0.82 (0.58–1.16)	0.254
SSRI			1.69 (1.19–2.40)	0.003
SNRI			1.20 (0.62–2.32)	0.583
Mirtazapine			1.52 (1.05–2.18)	0.025

TCA: tricyclic antidepressant; SSRI: selective serotonin reuptake inhibitor; SNRI: serotonin–norepinephrine reuptake inhibitor; HR: hazard ratio; CI: confidence interval.

## Data Availability

The data used in this study are subject to strict privacy and confidentiality requirements and therefore cannot be made publicly available. Legal restrictions prohibit the sharing of the raw data with third parties. To support reproducibility, the complete list of ICD-10 and ATC code definitions used to derive all study variables, together with the analytical specifications, can be made available by the authors upon reasonable request.
